# Suggestive Therapeutics in Psychopathia Sexualis; with Especial Reference to Contrary Sexual Instinct

**Published:** 1895-10

**Authors:** 


### Book Notices.

SUGGESTIVE THERAPEUTICS IN PSYCHOPATHIA SEXUALIS; WITH ESPECIAL REFERENCE TO CONTRARY SEXUAL INSTINCT. By Dr. A. Von Schrenck-Notzing (Munich, Germany). Authorized translation from the German by Charles Gilbert Chaddock, M. D., Professor of Diseases of the Nervous System, Marion-Sims College of Medicine, St. Louis; member of the American Medico-Psychological Association; Attending Neurologist to the Rebekah Hospital, St. Louis, Mo., etc., etc. One volume, Royal Octavo, 325 pages. Extra Cloth, $2.50 net; Sheep, $3.50 net. Sold only by subscription to the medical profession exclusively. Philadelphia: The F. A. Davis Co., Publishers, 1914 and 1916 Cherry St.

In dealing with questions of psychopathy it requires a clear mind and a strong sense of moral proprieties to produce a work the acceptance of which shall depend upon the usefulness of the book rather than upon the sensually perverted thoughts excited in the minds of the readers. Sexual excitations, debilities and perversions are, when discussed in detail, at best but a foul mess; and a work should have a more distinct raison d 'etre than merely that of calling attention to the subject, and thus stirring up the filth. A very large proportion of the recent additions to the literature of the subject given the profession and the public are in the writer's humble opinion, entirely unnecessary and absolutely harmful when permitted widespread presentation, as in a manner condoning as disease faults of development and education, and even exciting by suggestion similar faults in impressionable natures. It can not well be proved, but it is highly probable, that the recent great increase in recognized sexual perversion is by no means entirely due to an increased scientific interest on the part of physicians in the subject, but is at least to some degree, the direct product of the influence of sexual literature upon persons of sensual natures and weak moral inhibitive powers. Such literature is eagerly sought by a large class of the public, and it has unfortunately been but too easy for the laity to obtain such publications at will. A record of one case of sexual perversion, written not in the bare exactness of scientific medicine, but sensationally attractive because of its bawd-like descriptions and details of lasciviousness, becomes a strong suggestive motive to the weaklings who crave such excitement; and, with the idea of the irresponsibility of disease further engrafted, provokes them to licentiousness.

Yet it probably is necessary that at least occasionally the compost-heap be searched over; there may be an occasional object of value that can be saved; but for the sake of public and private decency, to avoid as much as possible the degeneracy in matters sexual and moral that seems impending, let us have as little publicity as possible; let us have that little which is worth saying said briefly and done.

The work of Von Schrenck-Notzing in these particulars is worthy of much commendation. It is rather prolix in the details of some of its histories, permitting quite too much of the nauseating minutiæ; and this seems a distinct fault, the avoidance of which would have made the book cleaner and better. The author is well to the fore among sexual psychologists, however, in insisting upon the educational factor in all forms of sexual psychopathy. He does not deny the possibility of an hereditary influence, but believes this transmitted condition to be simply a predisposition, a state of low general nervous and moral inhibition, a neurasthenia. While it is not held impossible that cases of sexual perversion, depression or excitation may be directly transmitted hereditarily, yet none such have as yet beyond dispute been reported; and in most instances the influence of sexual maleducation may be demonstrated, acting either with or without previous neurasthenic predisposition. Such a view of the responsibility of the sexual pervert, leading to a legal recognition of acts and his punishment for them is likely to bring about more actual good to the community than endless efforts toward amelioration of the evil as a disease. The matter may thus well be compared with the evil of alcoholic intemperance—it may eventually by persistent practice become a disease, but it is voluntarily assumed, and the responsibility can not be evaded, nor the punishment avoided. Severe punishment, as in the recent Wilde case, is likely to be productive of good and preventive of at least open perversion among large numbers and for a long time. The author is to be congratulated upon his decided and reasonable position on this phase of the question.

Another feature in which the work in hand is distinctly in advance, exists in its endeavors to simplify the classification and nomenclature of the subject. There are accepted by the author three types—*sexual hyperæsthesia* (excitation in all its excessive forms), *sexual anæsthesia* (depression in its abnormalities), and *sexual paræsthesia* (all forms of sexual perversions). The terms "sadism" and "masochism," adopted from the names of popular writers, in whose writings characters of the corresponding forms of perversion were described, are replaced by the word "algolagny," a compound of two Greek words, signifying pain and sexual lust. Thus "sadism," sexual excitement or satisfaction through or accompanied by acts of cruelty to the object of the love, becomes "active algolagny;" while "passive algolagny" is intended to convey the idea of sexual gratification with or by cruelty inflicted upon the pervert himself. Such innovations as these and others are directly in the line of improvement, simplifying a classification which had become burdened with a lot of almost meaningless and insignificant differences.

The main purpose of the book is, however, its greatest justification, the demonstration of the possibility of cure even among the most pronounced and confirmed perverts. The method of cure, hypnotic suggestion, may tend to destroy the value of the book among the mass of physicians, few of whom feel able or care to attempt it, because of their ignorance of the hypnotic influence and its technique. That this is a mistake will probably be demonstrated by time and growing experience in the many lines in which hypnotism is being employed in therapeusis. Among physicians, by habit and necessity exercising constantly their powers of suggestion and will, few are without sufficient force to produce a hypnotic suggestion; and a proper understanding of the real nature of the practice and its phenomena will quickly determine many a successful trial in this field. It is a widespread error to believe actual sleep, catalaps and post-hypnotic amnesia are necessary features of each case; on the contrary, they are rather the exception, decided influence being almost always possible in sentient states of the subject. The large proportion of cures and improvements detailed in von Schrenck-Notzing's work amply prove the value of the procedure in these functional anomalies.

Dr. Chaddock's work has been well done; aside from a few

Germanisms and several other minor errors of expression, the translation is blameless. It is a relief, too, to know that the publishers recognize the propriety of limiting the sale of the book to the medical profession; they thus avoid what has seemed to many a mistake in the distribution of von Kraft-Ebing's work in its American edition, its sale among numbers whose only desire for it was a prurient curiosity. A. J. S.

